# Optimization of Diamond Polishing Process for Sub-Nanometer Roughness Using Ar/O_2_/SF_6_ Plasma

**DOI:** 10.3390/ma18112615

**Published:** 2025-06-03

**Authors:** Lei Zhao, Xiangbing Wang, Minxing Jiang, Chao Zhao, Nan Jiang, Kazhihito Nishimura, Jian Yi, Shuangquan Fang

**Affiliations:** 1 School of Mechanical Engineering, Yangzhou University, Yangzhou 225009, China; zhaolei@nimte.ac.cn (L.Z.); wangxiangbing@nimte.ac.cn (X.W.); 2 Ningbo Institute of Materials Technology and Engineering, Chinese Academy of Sciences, Ningbo 315201, China; jiangminxing@nimte.ac.cn (M.J.); jiangnan@nimte.ac.cn (N.J.); nishimura@cc.kogakuin.ac.jp (K.N.); 3 School of Material Science and Engineering, Guilin University of Electronic Technology, Guilin 541004, China; 4 School of Chemical Engineering, Yangzhou University, Yangzhou 225009, China; mz120240780@stu.yzu.edu.cn

**Keywords:** inductively coupled plasma, single-crystal diamond, roughness, atomic force microscopy, process parameters

## Abstract

Diamond, known for its exceptional physical and chemical properties, shows great potential in advanced fields such as medicine, semiconductors, and optics. However, reducing surface roughness is critical for enhancing its performance. This study employs inductively coupled plasma (ICP) polishing to etch single-crystal diamond and analyzes the impact of different etching parameters on surface roughness using atomic force microscopy (AFM). Using the change in surface roughness before and after etching as the main evaluation metric, the optimal etching parameters were determined: Ar/O_2_/SF_6_ gas flow ratio of 40/50/10 sccm, ICP power of 200 W, RF bias power of 40 W, chamber pressure of 20 mTorr, and etching time of 10 min. Results show that increased etching time and SF_6_ flow rate raise surface roughness; although higher ICP and RF power reduce roughness, they also cause nanostructure formation, affecting surface quality. Lower chamber pressure results in smaller roughness increases, while higher pressure significantly worsens it. Based on the optimized process parameters, the pristine single-crystal diamond was further etched in this study, resulting in a significant reduction of the surface roughness from 2.22 nm to 0.562 nm, representing a 74.7% decrease. These improvements in surface roughness demonstrate the effectiveness of the optimized process, enhancing the diamond’s suitability for high-precision optical applications.

## 1. Introduction

Diamond is an attractive functional material due to its excellent mechanical, electrical, chemical, and thermal properties that far exceed those of many other materials. It can be used in a wide range of applications, including medicine, optical devices, biosensors, quantum computing, potentially microelectronics and microelectromechanical systems, as well as next-generation wide-bandgap semiconductor electronics [1,2,3,4,5]. One of the most remarkable properties of diamond is its stability, which allows it to remain stable in harsh environmental conditions such as abrasion, radiation, chemical corrosion, high temperatures and high pressures [6,7]. With the continuous development of science and technology, the requirements for diamond materials are getting higher and higher, especially in terms of performance, size and surface roughness. The ultra-high hardness and excellent mechanical properties of diamond make it difficult to be processed by ordinary machining methods, limiting its wide application. Traditionally, methods to reduce diamond surface roughness have relied mainly on grinding and polishing. However, microcracks or other damages may be introduced to the diamond’s surface during the grinding and polishing process [8,9], which in turn affects its performance and reliability. In addition, grinding and polishing diamond to bring the surface roughness to the sub-nanometer level requires a lot of labor, material and time, resulting in high costs. Therefore, there is an urgent need to explore other technological means to achieve rapid polishing of diamond surfaces. Plasma etching technology, which has the advantages of high etching rate, good selectivity, etching anisotropy, low linewidth ratio, high surface accuracy, and controllable etching, is the most likely key technology to realize the rapid polishing of diamond surfaces [10,11,12].

Reactive ion etching technology is a plasma etching method that selectively removes material in a plasma environment through the synergistic effect of chemical reaction and physical sputtering to achieve efficient and high-precision etching, which is widely used in precision structuring of diamond because of its wide etching area and excellent uniformity. Pure oxygen plasma etching process has been successfully applied in the production of diamond micromechanical components, while most of the etching processes are based on the combination of oxygen plasma and auxiliary gases [13,14]. Ar, SF_6_, Cl_2_ or CF_4_ are added to the oxygen plasma to improve the surface quality, but these gases affect the etching rate and etching anisotropy [15,16,17,18,19,20], among which the most commonly used reactive gases include O_2_, Ar, CF_4_, and SF_6_. Depending on different combinations of the gas compositions, sub-nanometer roughness surfaces can be achieved for diamond. According to the available literature, graphite or amorphous carbon can be etched faster than diamond [21,22], which explains the significant increase in the etching rate with the addition of argon. Not only this, but the formation of a flatter etched surface can be facilitated by introducing a small amount of fluorine-based auxiliary gases into the gas mixture. To date, a large number of studies have been reported on the rapid polishing of diamond surfaces using reactive ion etching [14,23,24,25]. For example, Jiarong Yu et al. [16] used inductively coupled plasma etching-assisted single-crystal diamond polishing to reduce surface roughness to an Ra value of 0.185 nm over a 10 μm × 10 μm range, significantly improving surface quality and laying a foundation for further diamond surface treatment advancements. In contrast, our study further optimizes the process parameters, reducing roughness from 2.22 nm to 0.562 nm, demonstrating the effectiveness of the optimized process and enhancing the potential for high-precision optical applications. Many researchers have investigated one or two gas mixtures to achieve diamond surface roughness reduction. Xinyu Li et al. [15] employed argon and oxygen plasma in combination with other techniques to polish polycrystalline diamonds, achieving remarkable results with surface roughness as low as 0.16 nm; however, there are few studies on whether the three gas mixtures of SF_6_, O_2_, and Ar can achieve sub-nanometer surface roughness on single-crystal diamond. Therefore, it is of great significance to study the effect of the reactive ion etching process of these three gas mixtures on the surface roughness of single-crystal diamond.

In this paper, we focus on the effect of inductively coupled plasma (ICP) process parameters based on Ar/O_2_/SF_6_ gas mixture on the surface roughness of single-crystal diamond. We focused on the effects of parameters such as ICP power, RF power, gas ratio (Ar/O_2_/SF_6_), chamber gas pressure, and etching time on the surface roughness, and finally determined a set of optimal process parameters. Although the surface quality enhancement after etching is relatively small, this improvement is informative for future surface treatment processes in precision instrument manufacturing. We believe that this study provides a feasible range of parameters for optimizing the surface treatment of diamond and lays the foundation for further research in related fields in the future.

## 2. Experimental

All samples used in the experiment were laboratory-synthesized 3 × 3 × 0.5 mm^3^ (100) type IIa single-crystal diamonds. Prior to the experiment, the samples were cleaned by immersing them in boiling piranha solution (H_2_SO_4_:H_2_O_2_ = 7:3) for 8 h. They were then thoroughly rinsed in acetone and anhydrous ethanol, followed by a nitrogen gas purge to ensure cleanliness. The diamond wafers used in this experiment were initially ground and polished by the supplier, achieving a roughness range of 1–10 nm. The Atomic Force Microscope (AFM) used for the experiments was a Bruker Dimension Icon, operating in tapping mode with a scan area of 20 × 20 μm^2^. The scan resolution was set to 512 data points per line, and the scanning rate was 0.5 Hz. The surface roughness was analyzed using NanoScope Analysis software (Dimension lcon), and the data were plotted using Origin software (OriginPro 2025b). NanoScope Analysis was employed to acquire and analyze the surface roughness, while Origin was used for plotting the resulting data. A 20 × 20 μm^2^ area was selected for analysis, consisting of the sample’s center and four corners, each located 0.5 mm away from their respective adjacent edges. These same locations were measured both before and after etching. Measurements were taken at these same locations before and after etching and the scanning direction was kept consistent by cycling the scan from top to bottom, left to right, and then left. The final surface roughness value was calculated as the average of these measurements, and the standard deviation was used to indicate the variation in roughness across the selected areas. The experiments were conducted using an inductively coupled plasma (ICP) device. The ICP system utilizes a high-frequency power supply (13.56 MHz) to energize an inductive coil, exciting electrons within the gas. These high-energy electrons collide with gas molecules, generating ions, electrons, and neutral particles, thereby forming a high-density plasma. Under the influence of an electric field, ions in the plasma are accelerated and bombard the sample surface, transferring kinetic energy and triggering a series of physical and chemical reactions. To ensure consistent conditions during each etching process, we chose a silicon substrate as the experimental material and placed the samples in the center of the substrate to ensure that the samples were always in the densest region of the plasma. This maximizes the consistency of the ion and particle energies to which each sample is exposed during the etching process. In addition, we controlled the experimental temperature to ensure that it was between 30 and 35 °C to avoid temperature fluctuations from affecting the etching results. With this setup, we were able to ensure that all samples were etched under the same experimental conditions, thus ensuring reproducible and reliable results.

The experiment consists of five parts.

Experiment I: This experiment aimed to investigate the effect of different etching times on the surface roughness of single-crystal diamond. The etching time was varied between 10 and 30 min, while other parameters were kept constant: ICP power at 200 W, RF power at 40 W, gas flow ratios of Ar (40 sccm), O_2_ (50 sccm), and SF_6_ (10 sccm), and chamber gas pressure at 20 mTorr. The detailed etching parameters are shown in Table 1. At the conclusion of the experiment, the optimal etching time for achieving the lowest surface roughness on the single-crystal diamond was determined through surface roughness measurements and analysis.

Experiment II: Building on the optimal etching time determined in Experiment I, this experiment further investigated the effect of different gas ratios on the surface roughness of single-crystal diamond. In this experiment, ICP power (200 W), RF power (40 W), and chamber air pressure (20 mTorr) were kept constant, while the gas ratio was varied. The specific etching parameters are shown in Table 2. At the end of the experiment, the optimal gas ratio for achieving the lowest surface roughness on the single-crystal diamond was determined through surface roughness measurements and analysis.

Experiment III: Building on the optimal etching time and gas flow rate determined in Experiments I and II, this experiment further investigated the effect of different ICP power levels on the surface roughness of single-crystal diamond. In this experiment, RF power (40 W) and chamber gas pressure (20 mTorr) were kept constant, while ICP power was varied. The specific etching parameters are shown in Table 3. At the conclusion of the experiment, the optimal ICP power for achieving the lowest surface roughness on the single-crystal diamond was determined through surface roughness measurements and analysis.

Experiment IV: Building on the optimal etching time, gas flow rate, and ICP power determined in Experiments I, II, and III, this experiment further investigated the effect of varying RF power on the surface roughness of single-crystal diamond. In this experiment, chamber gas pressure (20 mTorr) was kept constant, while RF power was varied. The specific etching parameters are shown in Table 4. At the conclusion of the experiment, the optimal RF power for achieving the lowest surface roughness on the single-crystal diamond was determined through surface roughness measurements and analysis.

Experiment V: Building on the optimal etching time, gas flow rate, ICP power, and RF power determined in Experiments I, II, III, and IV, this experiment further investigated the effect of varying chamber gas pressure on the surface roughness of single-crystal diamond. In this experiment, only the chamber gas pressure was varied, and the specific etching parameters are shown in Table 5. At the conclusion of the experiment, the optimal chamber gas pressure for achieving the lowest surface roughness on the single-crystal diamond was determined through surface roughness measurements and analysis.

## 3. Results and Discussion

### 3.1. Experiment on the Effect of Etching Time on Surface Roughness

**Figure 1:**
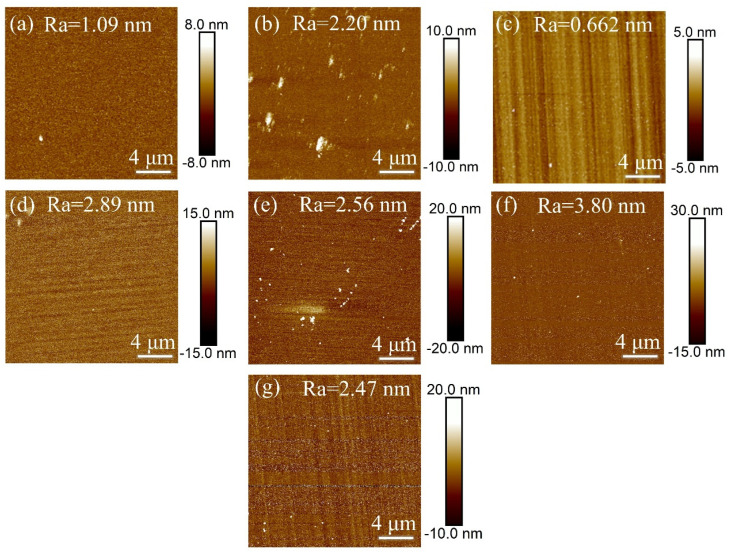
AFM images of single-crystal diamond surface roughness for different etching times: (**a**) 1 min, (**b**) 5 min, (**c**) 10 min, (**d**) 15 min, (**e**) 20 min, (**f**) 25 min, (**g**) 30 min.

In Experiment I, inductively coupled plasma (ICP) technology was used to investigate the effect of different etching times on the surface roughness of single-crystal diamond samples. The goal was to determine the optimal etching time. After reaching the specified etching times, the surface roughness of the samples was measured, with the results shown in Figure 1.

**Figure 2:**
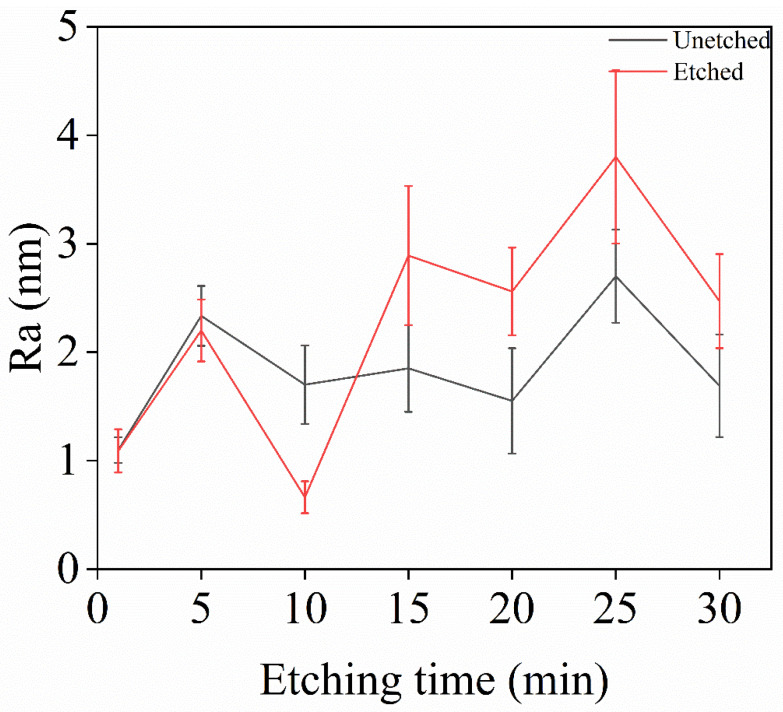
Roughness variation of single-crystal diamond before and after etching for different etching times.

Figure 2 shows the error bar graph of surface roughness for single-crystal diamond before and after etching at different etching times. It can be seen from the figure that the etching time between 1 and 10 min reduces the surface roughness of the diamond. At 1 min and 5 min the decrease in surface roughness after etching is very low due to the short etching time. However, as the etching time increases, the surface roughness after etching becomes higher compared to before etching. This can be attributed to the following process: during the short etching period (10 min), O_2_ promotes the oxidation of diamond, causing carbon on the diamond surface to react with O_2_ to form volatile compounds like CO and CO_2_. The weak anisotropy caused by F- ions effectively removes microscopic bumps, flattening the surface and reducing the roughness. As the etching time increases (15–30 min), the F- and O ions from O_2_ and SF_6_ decomposition increase and the reaction with the diamond surface carbon intensifies; the anisotropy [26] promoted by F^-^ is accompanied by plasma bombardment and chemical etching enhancement leading to an increase in the unevenness of the etching of some parts of the diamond surface, and hence an increase in the number of surface protrusions and thus an increase in the number of Diamond surface roughness after etching. From the AFM images in Figure 1a–g, it can be seen that the surface roughness also decreases at 5 min but not as much as at 10 min and the roughness at 10 min is as low as 0.662 nm. As the etching time increases, white nanopillar-like protrusions become apparent on the diamond surface, increasing the inhomogeneity and roughness of the etching.

In Figure 2 we can clearly see that the etching times of 1–15 min on the surface to reduce the roughness are reduced. In order to further confirm whether 10 min is the optimal etching time, this part of the experiment carried out the experimental design of 1–15 min, with every interval of 2 min on the samples to be etched, and the specific results of the experiment, as shown in Table 6. From the table, when the etching time is 1–5 min, the surface roughness does not change much because the plasma inside the chamber is not stabilized or just out of the stable state, and the diamond surface is not completely etched. At 5–10 min, the plasma reaches a stable state, and the chamber gas has enough time to react effectively with the diamond, so the roughness of the diamond surface is effectively reduced. However, as the etching time increases, the diamond surface deteriorates due to the intensification of the plasma bombardment and chemical etching reaction, resulting in an increase in the surface roughness. According to the results in the table, the optimum etching time is 10 min, and the decrease in surface roughness after etching under this time is the largest.

### 3.2. Experiments on the Effect of Ar/O_2_/SF_6_ Gas Flow Ratio on Surface Roughness

**Figure 3:**
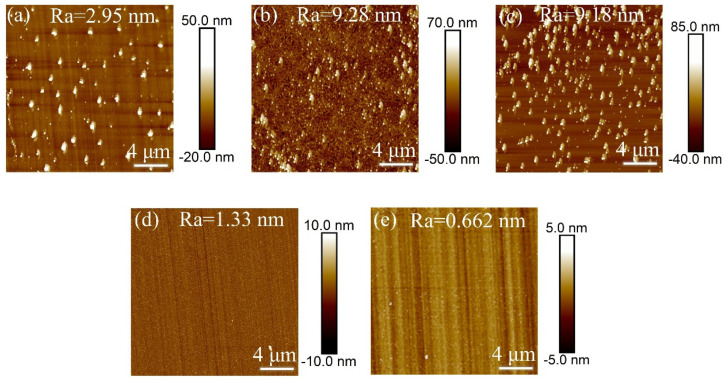
AFM images of single-crystal diamond surface roughness for different Ar/O_2_/SF_6_ gas flow ratios (**a**) 40/10/50 sccm, (**b**) 40/20/40 sccm, (**c**) 40/30/30 sccm, (**d**) 40/40/20 sccm, and (**e**) 40/50/10 sccm.

In Experiment II, inductively coupled plasma (ICP) technology was used to investigate the effect of different gas flow rate ratios on the surface roughness of single-crystal diamond samples during the etching process, with the goal of determining the optimal gas flow rate ratio. After the set etching time was reached, the surface roughness of the samples was measured, and the resulting surface roughness data is shown in Figure 3.

**Figure 4:**
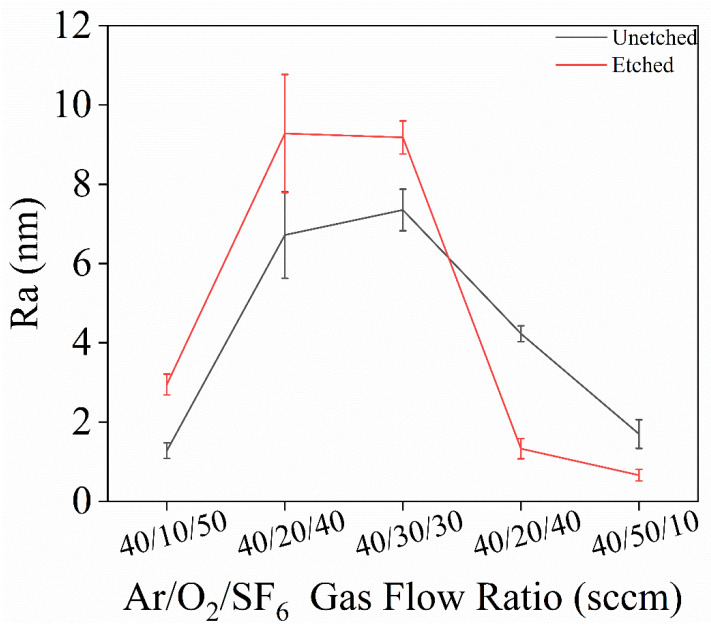
Roughness variation of single-crystal diamond before and after etching for different Ar/O_2_/SF_6_ gas flow ratios.

Figure 4 shows the error bar graphs of surface roughness for single-crystal diamond before and after etching at different gas flow ratios. From the figure, it is evident that when O_2_ increases and SF_6_ decreases, the surface roughness of the diamond changes. Specifically, when the Ar/O_2_/SF_6_ ratios are 40/40/20 and 40/50/10 sccm, the surface roughness of the diamond decreases, while the roughness increases for the other three gas ratios. This is because the increase in O_2_ effectively promotes the reaction between carbon and oxygen on the diamond surface, allowing for a more complete and uniform etching process, which reduces surface roughness. Additionally, reduced flow of sulfur hexafluoride gas reduces the F^-^ ions in the chamber and thus reduces their chemical etching and anisotropic effect on the diamond surface, allowing O_2_ etching to dominate and promoting surface oxidation reactions to make the surface smoother. From the AFM images in Figure 3a–e, it can be clearly seen that the surface roughness is minimized when the Ar/O_2_/SF_6_ gas flow rate is 40/50/10 sccm. At higher SF_6_ gas concentrations, sulfur hexafluoride reacts with the diamond surface to generate fluorocarbon compounds, which enhances the chemical etching effect. In addition, the oxidation of the diamond surface is weakened when the oxygen content is low, which does not allow the effective removal of the surface carbon and thus reduces the surface smoothing, which exacerbates the surface roughness of the etched surface. Therefore, in this study, an Ar/O_2_/SF_6_ ratio of 40/50/10 sccm is determined as the optimal gas flow rate ratio, resulting in fewer surface protrusions and better uniformity and surface quality after etching.

### 3.3. Experiments on the Effect of ICP Power on Surface Roughness

**Figure 5:**
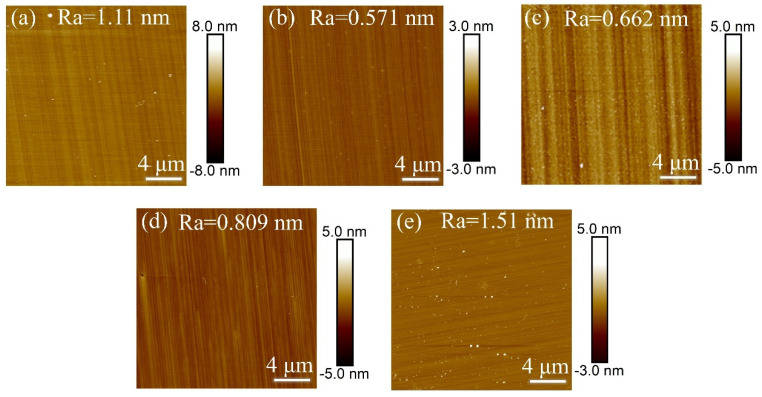
AFM images of single-crystal diamond surface roughness for different ICP powers (**a**) 100 W, (**b**) 150 W, (**c**) 200 W, (**d**) 250 W, and (**e**) 300 W.

In Experiment III, inductively coupled plasma (ICP) technology was used to investigate the effect of ICP power on the surface roughness of single-crystal diamond samples during the etching process, with the aim of determining the optimal ICP power. After the set etching time was reached, the surface roughness of the samples was measured, and the resulting surface roughness data is shown in Figure 5.

**Figure 6:**
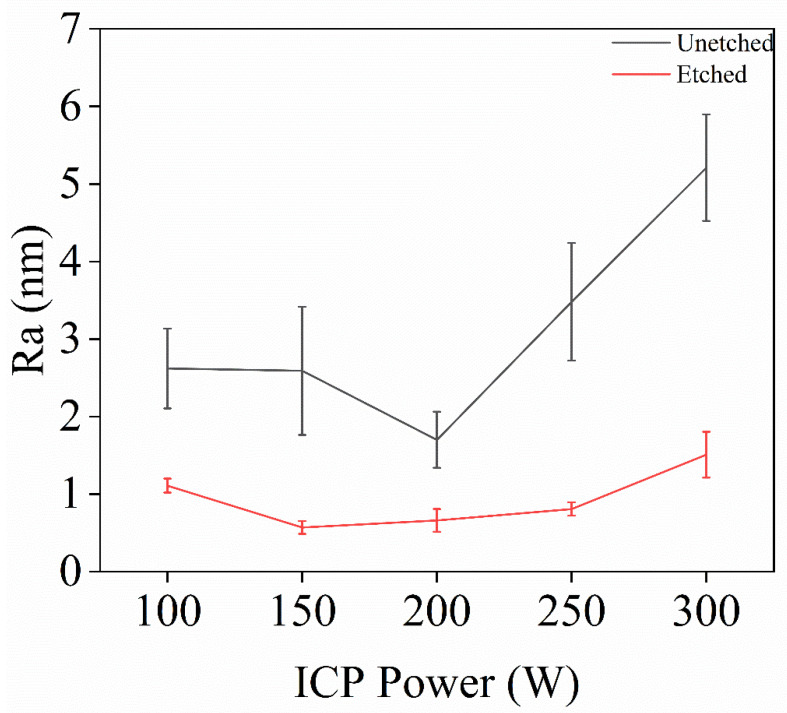
Variation of single-crystal diamond before and after etching for different ICP powers.

Figure 6 shows the error bar graph of surface roughness before and after etching of single-crystal diamond at different ICP power levels. Under varying ICP power, the surface roughness decreases after etching. This is because, during the ICP etching process, the ICP power primarily regulates ion energy. An appropriate increase in power enhances the bombardment effect of argon ions, strengthens the physical etching effect, and effectively removes rougher areas on the diamond surface. Additionally, higher ICP power activates the chemical reaction activity of O_2_, promoting the formation of C-O bonds and improving etching uniformity. In addition, the appropriate ICP power can regulate the plasma density and energy distribution during the etching process, improve the uniformity of the surface reaction, and thus enhance the quality of the etched surface. When the ICP power is appropriate, it can effectively enhance the oxidation of O_2_, promote the removal of carbon from the diamond surface, and then improve the smoothness of the surface. Meanwhile, SF_6_ can reduce the surface inhomogeneity caused by excessive etching at lower power. From the AFM images in Figure 5a–e, it is clear that the surface roughness decreased by 1.51 nm, 2.019 nm, 3.568 nm, 2.671 nm, and 3.7 nm, respectively, compared to before etching at different ICP power levels. This indicates that optimizing ICP power is crucial for improving the surface quality of diamond. Therefore, an ICP power of 200 W was selected, as it effectively reduces diamond surface roughness by balancing chemical etching and physical sputtering.

### 3.4. Experiments on the Effect of RF Power on Surface Roughness

**Figure 7:**
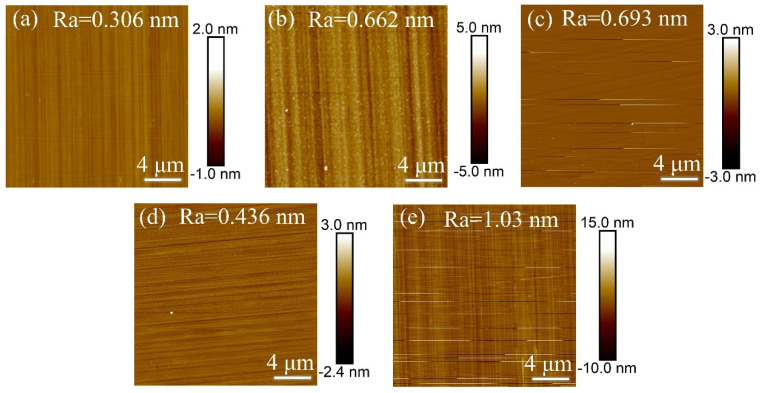
AFM images of single-crystal diamond surface roughness for different RF powers (**a**) 20 W, (**b**) 40 W, (**c**) 60 W, (**d**) 80 W, and (**e**) 100 W.

In Experiment IV, inductively coupled plasma (ICP) technology was used to investigate the effect of RF power on the surface roughness of single-crystal diamond samples during the etching process, with the aim of determining the optimal RF power. After the set etching time was reached, the surface roughness of the samples was measured, and the resulting surface roughness data is shown in Figure 7.

**Figure 8:**
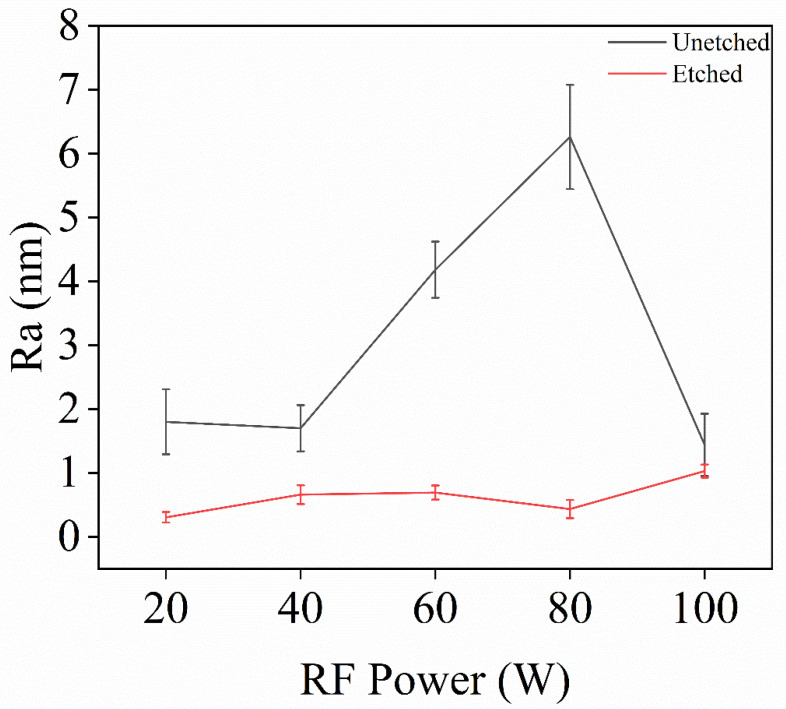
Roughness map changes before and after etching of single-crystal diamond for different RF powers.

Figure 8 presents the error bar graphs of surface roughness for single-crystal diamond before and after etching under different RF power levels. The results show that surface roughness is reduced after etching across all RF power settings, with RF power having a direct impact on ion kinetic energy and their bombardment effect on the diamond surface. At lower RF powers of 20 W and 40 W, ions possess lower velocity and kinetic energy, resulting in gentler bombardment. This allows for more uniform material removal and improved surface quality, maintaining a relatively smooth surface after etching. With the further increase in RF power, the density of the plasma and the kinetic energy of the ions increased, leading to intensified ion bombardment. The high-energy ions enhance the reaction between the surface and the gas, especially the chemical reaction between O_2_ and SF_6_, which makes the diamond surface etching reaction intensified. In this case, the role of O_2_ is more obvious, which effectively removes carbon from the diamond surface, while the F ions provided by SF_6_ enhance the etching reaction. With the further increase in RF power, too high energy may lead to inhomogeneous etching of localized areas on the diamond surface during the etching process, forming nanopillar structures and thus increasing the surface roughness. From the AFM images in Figure 7a–e, it is evident that the best surface quality is achieved at 20 W and 40 W. In contrast, at 60 W, 80 W, and 100 W, the presence of nanopillar protrusions and flake-like structures due to intensified anisotropic etching degrades the surface uniformity. Therefore, an RF bias power of 40 W is identified as the optimal setting, balancing surface quality and roughness reduction effectively.

### 3.5. Experiments on the Effect of Chamber Air Pressure on Surface Roughness

**Figure 9:**
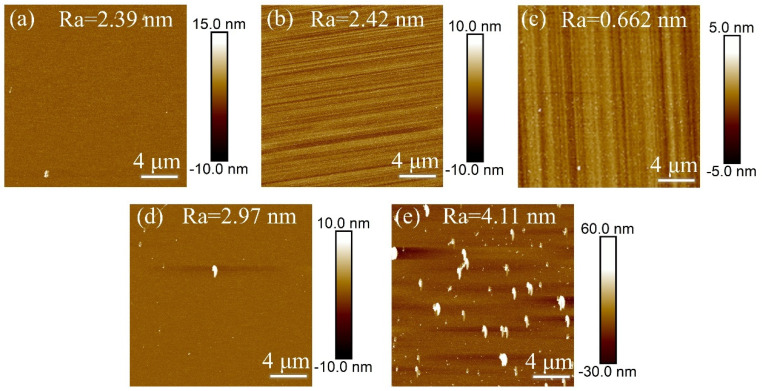
AFM images of single-crystal diamond surface roughness for different chamber air pressures (**a**) 10 mTorr, (**b**) 15 mTorr, (**c**) 20 mTorr, (**d**) 25 mTorr, (**e**) 30 mTorr.

In Experiment V, inductively coupled plasma (ICP) technology was used to investigate the effect of chamber air pressure on the surface roughness of single-crystal diamond samples during the etching process, with the goal of determining the optimal chamber air pressure. After the set etching time was reached, the surface roughness of the samples was measured, and the resulting surface roughness data is shown in Figure 9.

**Figure 10:**
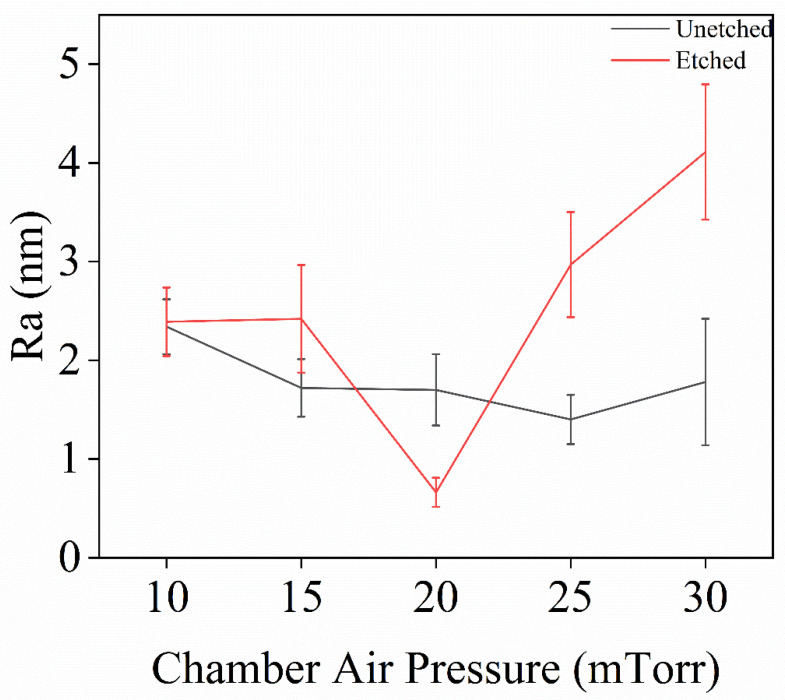
Roughness variation of single-crystal diamond before and after etching for different chamber air pressures.

Figure 10 shows the error bar graphs of surface roughness for single-crystal diamond before and after etching under different chamber air pressures. When the chamber air pressure is 20 mTorr, the surface roughness of the diamond decreases after etching, while the roughness increases under the other four air pressure conditions (10, 15, 25, and 30 mTorr). This behavior is primarily due to the impact of chamber air pressure on both chemical etching and physical bombardment. At low air pressures (10 and 15 mTorr), plasma density decreases, and the concentration of active particles, such as F and O, is insufficient. This leads to uneven chemical etching and slower etching in localized areas, causing the formation of white columnar protrusions and increasing surface roughness. At high gas pressures (25, 30 mTorr), the collision frequency of the gas increases and the mean free range of the ions in the plasma is shortened, which leads to more collisions of the ions with the gas molecules during transport In addition, the energy loss of the ions increases, and the bombardment energy of the Ar⁺ ions, in particular, decreases. Due to the reduced kinetic energy of the ions, the efficiency of surface etching decreases and the etching process becomes inhomogeneous. At this time, the reaction efficiencies of O_2_ and SF_6_ were also affected, which could not uniformly remove the carbon from the diamond surface, leading to enhanced localization of the surface reaction and further formation of irregular nanopillar structures. These factors work together to increase surface roughness. At 20 mTorr, an optimal balance between chemical etching and ion bombardment is achieved. This pressure ensures sufficient F and O participation in uniform etching while maintaining the appropriate Ar⁺ bombardment energy, resulting in more uniform surface etching and a reduction in roughness. As shown in the AFM images in Figure 9a–e, at 10 and 15 mTorr, uneven etching leads to significant nanopillar formations, increasing surface roughness. At 25 and 30 mTorr, wider nanopillars appear, further increasing roughness due to high-pressure etching. However, at 20 mTorr, the etched surface quality is superior, with reduced roughness. Therefore, 20 mTorr is identified as the ideal air pressure for optimizing etching uniformity and surface quality.

### 3.6. Optimal Process Results Etching

**Figure 11:**
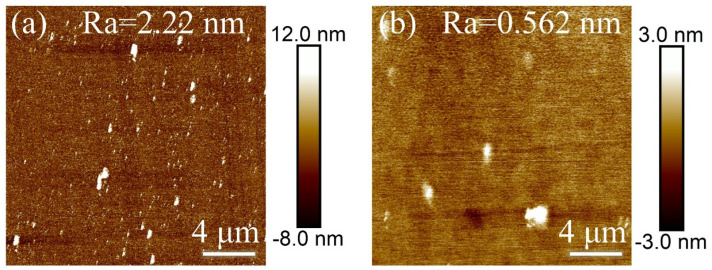
Optimal process etching (**a**) surface roughness in the range of 20 × 20 μm^2^ of single-crystal diamond before and (**b**) after etching.

Through an in-depth analysis and comparison of five sets of experiments, this study successfully optimized and determined the best process parameters for diamond polishing using inductively coupled plasma (ICP): ICP power of 200 W, RF power of 40 W, Ar/O_2_/SF_6_ gas flow rate of 40/50/10 sccm, chamber air pressure of 20 mTorr, and an etching time of 10 min. Based on these optimized parameters, the pristine diamond was further etched to evaluate the surface quality and assess whether sub-nanometer roughness could be achieved. As shown in Figure 11, the surface roughness of the etched diamond was successfully reduced to 0.562 nm under the optimal process conditions, achieving a sub-nanometer polishing effect. In comparison, the surface roughness of the diamond after merchant polishing, before etching, was 2.22 nm, accompanied by numerous nanopillar protrusions. After the optimized etching process, the surface roughness was significantly reduced, and the nanopillar protrusions were notably diminished, further proving the reliability of inductively coupled plasma polishing for diamond.

## 4. Conclusions

In this study, the inductively coupled plasma (ICP) etching technique was systematically used to investigate the effects of various ICP process parameters (ICP power, RF power, gas flow rates, chamber air pressure, and etching time) on the surface roughness of single-crystal diamond. The experimental results show that, under the optimized conditions, the surface roughness (Ra) of single-crystal diamond can be reduced to 0.562 nm (20 × 20 μm^2^): ICP power of 200 W, RF power of 40 W, chamber air pressure of 20 mTorr, and a gas mixture of Ar/O_2_/SF_6_ (40/50/10 sccm), with an etching time of 10 min. These optimized parameters successfully reduce the surface roughness to the sub-nanometer scale while effectively minimizing nanopillar protrusions caused by mechanical grinding and polishing. The significant improvement in surface quality provides a promising approach for ultra-precision machining of diamond surfaces. Moreover, the findings lay a solid foundation for expanding the application of this technology into high-end fields such as optics and medicine. The results of this study not only pave the way for future developments in diamond surface processing but also highlight its potential for advanced applications in precision engineering.

## Figures and Tables

**Table 1:** Table of process parameters for Experiment I.

	ICP (W)	RF (W)	Ar (sccm)	O_2_ (sccm)	SF_6_ (sccm)	Chamber Air Pressure (mTorr)	Etching Time (min)
I	200	40	40	50	10	20	1
							5
							10
							15
							20
							25
							30

**Table 2:** Table of process parameters for Experiment II.

	ICP (W)	RF (W)	Ar (sccm)	O_2_ (sccm)	SF_6_ (sccm)	Chamber Air Pressure (mTorr)	Etching Time (min)
II	200	40	40	10	50		
			40	20	40		
			40	30	30	20	10
			40	40	20		
			40	50	10		

**Table 3:** Table of process parameters for Experiment III.

	ICP (W)	RF (W)	Ar (sccm)	O_2_ (sccm)	SF_6_ (sccm)	Chamber Air Pressure (mTorr)	Etching Time (min)
III	100	40	40	50	10	20	
	150						
	200						10
	250						
	300						

**Table 4:** Table of process parameters for Experiment IV.

	ICP (W)	RF (W)	Ar (sccm)	O_2_ (sccm)	SF_6_ (sccm)	Chamber Air Pressure (mTorr)	Etching Time (min)
IV		20	40	50	10	20	
		40					
	200	60					10
		80					
		100					

**Table 5:** Table of process parameters for Experiment V.

	ICP (W)	RF (W)	Ar (sccm)	O_2_ (sccm)	SF_6_ (sccm)	Chamber Air Pressure (mTorr)	Etching Time (min)
V			40	50	10	10	10
						15	
	200	40				20	
						25	
						30	

**Table 6:** Table of surface roughness variation with etching time.

Etching Time (min)	1	3	5	7	9	10	11	13	15
Unetched (nm)	1.10	1.88	2.34	2.28	1.87	1.7	1.86	1.24	1.85
Etching (nm)	1.09	1.79	2.20	1.99	1.10	0.662	1.97	1.57	2.89
Variation	0.9%	4.8%	5.9%	12.7%	41.2%	61.1%	−5.9%	−26.6%	−56.2%

## Data Availability

The original contributions presented in the study are included in the article, further inquiries can be directed to the corresponding authors.
